# Sepsis in a Patient With Atopic Dermatitis Following Upadacitinib Discontinuation

**DOI:** 10.7759/cureus.99567

**Published:** 2025-12-18

**Authors:** John A Gallagher, Diego Aragón-Caqueo, Arhum Mahmood, Steven Daveluy

**Affiliations:** 1 Department of Internal Medicine, Wayne State University School of Medicine, Detroit, USA; 2 Faculty of Medical Sciences, Universidad de Santiago de Chile, Santiago, CHL; 3 Department of Internal Medicine, Henry Ford Health System, Detroit, USA; 4 Department of Dermatology, Wayne State University, Detroit, USA

**Keywords:** atopic dermatitis, clinical dermatology, cost barriers, infectious cellulitis, infectious diseases, insurance barriers, jak inhibitors, patient support programs, sepsis, upadacitinib

## Abstract

Atopic dermatitis (AD) is a common chronic atopic skin condition. Severe AD may require treatment with systemic immunomodulation for disease control. Janus kinase (JAK) inhibitors such as upadacitinib are an increasingly prescribed class of immunomodulators effective for AD. However, cost and insurance coverage represent major barriers to accessing these medications. Disruptions of treatment with systemic immunomodulators predispose patients with AD to flares that can cascade into severe consequences, including sepsis. Therefore, maintenance of AD treatment is essential, and patient support programs (PSPs) offer a solution to ensure continuity of treatment. We present a case of a young adult with severe AD who lost insurance coverage and subsequently discontinued upadacitinib, leading to disease relapse complicated by cellulitis, sepsis, and acute liver failure. We highlight PSPs as an impactful solution to maintain access to AD therapy and prevent such complications in similar patients.

## Introduction

Atopic dermatitis (AD) is a common chronic inflammatory skin condition. The prevalence of AD in the United States (US) pediatric population is approximately 12.6%, and the disease is associated with high rates of atopic comorbidities and a negative impact on quality of life [1]. While the prevalence of AD in the US adult population is lower at approximately 7.3%, there is growing awareness that childhood AD may persist to adulthood [2]. Compared to AD in children, AD in adults more commonly presents with intermittent moderate-to-severe flares that are often inadequately controlled by topical therapies alone [3]. As a result, adults are more likely to require systemic therapy, primarily immunomodulators or immunosuppressants, to achieve disease control [4]. Janus kinase (JAK) inhibitors have revolutionized the management of moderate-to-severe AD in adolescents and adults since their introduction in 2020, offering a safe and effective option with rapid symptom relief and disease control [5]. Furthermore, they have long-term benefits in restoring skin barrier function, as they promote keratinocyte differentiation, enhance filaggrin expression, and upregulate essential skin barrier proteins and lipids, leading to substantial and sustained barrier restoration [6].

Although current clinical guidelines recommend the use of JAK inhibitors in patients with moderate-to-severe AD [7], high drug costs and insurance coverage represent barriers to access [8]. However, population-based studies in diverse healthcare settings suggest that JAK inhibitors might be a good value option, considering their efficacy and safety, especially in adults with severe AD requiring systemic immunomodulators [9-11]. Regarding treatment duration, there is no consensus on the optimal time to discontinue therapy once disease control is achieved. When de-escalation is considered, the strategy should be individualized and closely supervised by the treating clinician. Continuation of treatment in patients experiencing gaps or transitions in healthcare remains essential to maintain disease control and prevent serious complications. As AD impairs skin barrier function and the immune response to certain skin microbes such as *Staphylococcus aureus* (*S. aureus*), loss of disease control may predispose patients to serious infectious complications. *Staphylococcus​​​​​​ aureus* colonization rates in patients with AD are as high as 90%, approximately 10 times higher than the general population, with an increased risk of impetiginization and sepsis [12,13]. While the exact prevalence of serious infectious complications in patients with AD is unknown, a previous study found a significantly higher rate of infections (42.1%) in hospitalized patients with AD than in those without (25.4%) [13]. We present a case of a patient with severe AD who lost access to upadacitinib therapy and subsequently developed cellulitis with sepsis, leading to acute liver failure. The originality of this case lies in the complications arising from loss of access to therapy, which, in the context of JAK inhibitor use, remain scarcely documented in real-world scenarios. We highlight the importance of continuous therapy in patients with severe AD and offer solutions for clinicians to maintain therapy in patients with barriers to care.

## Case presentation

A 26-year-old man with a long-standing history of allergic rhinitis, asthma, pet/dust allergies, and atopic dermatitis (AD) presented to the emergency department (ED) with a two-week history of a skin rash with worsening pruritus. Notably, the patient lost insurance coverage upon turning 26 and aging out of his parents’ insurance plan, resulting in a nine-month lapse in healthcare. During this time, he discontinued upadacitinib, which was his maintenance therapy.

Regarding his history of AD, he was diagnosed as a child. Throughout his childhood, his AD was often unresponsive to topical steroids, and he experienced frequent flares that required multiple courses of oral corticosteroids. At the age of 22, he transitioned to dupilumab and continued it for over two years, achieving a partial clinical response. Subsequently, he transitioned to upadacitinib 15 mg daily, which led to near-complete skin clearance within 2-3 months, and maintained significant improvement for approximately one year. However, upon turning 26 and losing eligibility for his parents’ insurance coverage, he was unable to access upadacitinib for nearly nine months, ultimately resulting in an exacerbation of his AD.

Two months after discontinuing upadacitinib, he began experiencing flares that were partially managed using moisturizers, triamcinolone 0.1% cream, and a prednisone 60 mg taper for 21 days. During this time, he applied for health insurance and was waiting for approval. Approximately nine months after discontinuing the upadacitinib and six months after finishing his prednisone taper, the patient began to experience intense itching and exacerbation of eczema on his face, arms, legs, and trunk, which progressed over two weeks. The worsening eczema then led him to present to the ED.

**Figure 1:**
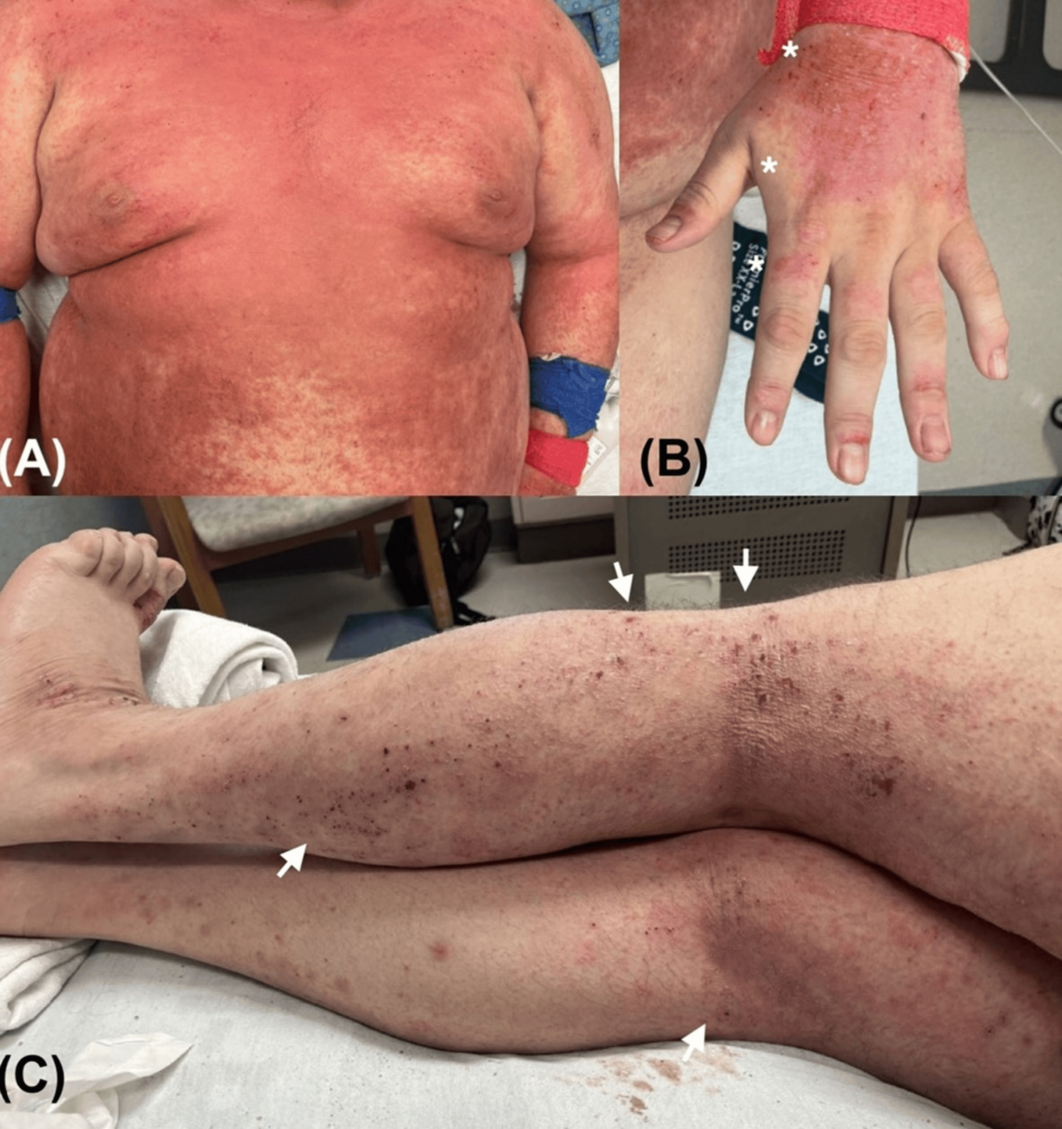
Presentation to the emergency department with flaring atopic dermatitis (A) Multiple erythematous papules coalescing into plaques with excoriations and edema of the trunk. (B) Dorsal hand with lichenified excoriated plaque (marked with *). (C) Multiple excoriated papules and lichenified plaques on the lower extremities, most prominent in the popliteal fossae (white arrows).

At presentation, physical examination revealed diffuse erythematous papules and lichenified scaly plaques involving the scalp, face, trunk, and all extremities (Figure 1). The leading diagnostic suspicion was a severe exacerbation of baseline atopic dermatitis with secondary infection. Additional differential diagnoses included contact dermatitis, morbilliform drug eruption, and pityriasis rubra pilaris, among others. The total Eczema Area and Severity Index (EASI) [14] score was 28.5, with regional sub-scores of 4.5 for the head and neck, 7.2 for the upper limbs, 9.6 for the trunk, and 7.2 for the lower limbs.

The face was markedly affected, with diffuse erythema and prominent eyelid edema. The conjunctivae showed bilateral hyperemia with associated mucopurulent discharge, while the oral mucosa was unaffected. The cervical folds were intensely involved, showing confluent erythematous papules and plaques with marked erythema and exudation, without areas of spared skin. On the upper trunk, widespread erythematous papules coalescing into plaques were noted, with a prominent exudative component interspersed with spared areas, particularly around the axillary folds and periumbilical region. The inguinal folds were also relatively spared, whereas the gluteal region displayed erythematous excoriated papules. The genital mucosa was unaffected.

The upper extremities showed diffuse erythematous papules and plaques with greater severity over the proximal arms and shoulders, while relative sparing was observed along the medial aspects of both arms. The dorsum of both hands exhibited more pronounced lichenification with crusted erosions, whereas the fingers were mostly spared, except for focal paronychia. The palms were preserved.

The lower extremities displayed confluent papules and plaques with a predominantly excoriated and lichenified pattern rather than an exudative one. The involvement was most pronounced along the knee folds, while the dorsum of the feet exhibited lichenified plaques with overlying crusted erosions. The soles were spared.

His vital signs were notable for borderline hypotension of 97/67 mm Hg, tachycardia of 128 beats per minute, a mildly elevated body temperature of 37.5°C, and tachypnea of 28 breaths per minute (Table 1). Laboratory workup showed leukocytosis (24.6 K/mcL), elevated lactate (6.5 mmol/L), elevated serum creatinine (3.82 mg/dL), and elevated liver function tests (LFTs) (alanine aminotransferase (ALT): 5,208 IU/L, aspartate transaminase (AST): 13,010 IU/L, bilirubin: 4.2 mg/dL, and prothrombin time/international normalized ratio (PT/INR): 37.4 seconds/3.5) (Table 2). Electrolytes showed hyponatremia (sodium: 133 mmol/L). Overall, the clinical picture was consistent with severe sepsis with associated end-organ liver failure and kidney injury [15]. Blood culture samples were obtained, and empiric broad-spectrum antibiotics (loading doses of vancomycin 2 g and ceftriaxone 2 g) with fluid resuscitation were initiated within hours of his presentation to the ED.

**Table 1:** Patient’s vital signs upon presentation Values in bold text are abnormal.

Vital sign	Patient value	Reference range
Blood pressure (mm Hg)	97/67	90/60-120/80
Pulse (beats per minute)	128	60-100
Temperature (°C)	37.5	36.5-37.3
Respiratory rate (breaths per minute)	28	12-20

**Table 2:** Patient’s pertinent laboratory values upon presentation Values in bold text are abnormal. ALT: alanine aminotransferase, AST: aspartate transaminase, PT: prothrombin time, INR: international normalized ratio

Laboratory test	Patient value	Reference range
White blood cells (K/mcL)	24.6	3.8-10.6
Lactate (mmol/L)	6.5	<2.1
Creatinine (mg/dL)	3.82	<1.28
ALT (IU/L)	5,208	<52
AST (IU/L)	13,010	<35
Bilirubin (mg/dL)	4.2	<1.2
PT (seconds)	37.4	11.5-14.5
INR	3.5	0.8-1.2
Sodium (mmol/L)	133	135-145

**Figure 2:**
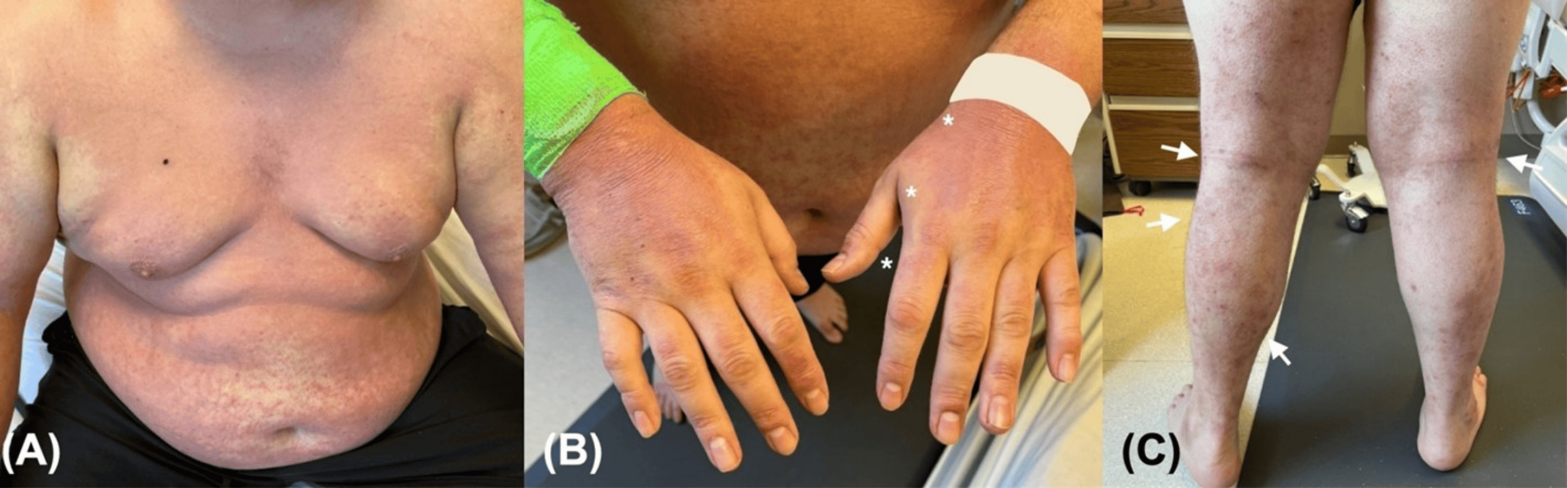
Clinical images three days after initiation of treatment (A) Marked reduction of erythema and edema of the upper trunk. (B) Improvement in excoriations, scaling, and lichenification of the dorsal hands (marked with *). (C) Significant reduction of lower extremity flexural involvement (white arrows).

After one day, his condition notably improved, with normalizing vital signs, down-trending liver enzymes, and recovering renal function. Antibiotics and fluids were continued. Prednisone 40 mg daily was introduced, along with triamcinolone 0.1% ointment two times daily and moist wound care with petrolatum. His skin involvement improved over a few days (Figure 2).

Blood cultures revealed gram-positive cocci in clusters that were catalase- and coagulase-positive on testing, consistent with *S. aureus*. Given the known association of uncontrolled AD with *S. aureus*, this suggested a cutaneous source of sepsis. Antimicrobial susceptibility testing showed the organism to be susceptible to cefazolin (4 μg/mL minimum inhibitory concentration (MIC)), daptomycin (1 μg/mL MIC), and oxacillin (0.25 μg/mL MIC), findings consistent with methicillin (oxacillin)-sensitive *S. aureus* (MSSA) [16]. Antibiotics were de-escalated to cefazolin based on these results. After five days of treatment, two sets of blood cultures remained persistently negative. The patient was discharged on linezolid 600 mg twice daily for 14 days for uncomplicated MSSA bacteremia [16]. He was also discharged on prednisone 40 mg daily for seven days. For long-term care of his AD, he had outpatient appointments with a primary care provider and dermatologist a few days later.

## Discussion

Our patient had a long history of severe AD with multiple past treatment failures. While upadacitinib provided effective disease control, the patient unfortunately experienced a gap in insurance coverage and lost access to the medication. As a result, he not only discontinued the drug but also lost the regular follow-up appointments essential for continuity of care. Pruritus worsens as early as four days after upadacitinib withdrawal, while skin clearance is typically lost within four weeks [17]. Although systemic corticosteroids can provide rapid flare suppression, they carry risks that often outweigh the benefits, including rebound flares and increased risk of infection [7]. Thus, current AD management guidelines recommend against routine systemic corticosteroid use, restricting them to short-term courses for acute exacerbations or as a bridge to steroid-sparing therapies [7,18]. It is likely that in our patient, discontinuation of upadacitinib for severe AD, in combination with a steroid rebound flare, predisposed him to an AD exacerbation and sepsis.

With adequate AD management, skin colonization by bacteria tends to be superficial and does not progress to systemic infection [12]. However, in the setting of inadequate disease control, an impaired skin barrier combined with the use of immunosuppressive medications can facilitate bacteremia and potential progression to sepsis, posing life-threatening complications. Although the association of sepsis and uncontrolled eczema is not novel [13], it remains a highly preventable complication important for clinicians to recognize.

This case reflects the costly nature of losing medical insurance, as young adults aged 19-34 make up the most uninsured group in the US [19], and such barriers can have detrimental effects for patients with AD taking costly treatments such as upadacitinib [20]. Patient support programs (PSPs) are structured services, typically sponsored by pharmaceutical companies or specialty pharmacies, that offer financial assistance to patients for prescription medications [21]. PSPs commonly assist patients who lack insurance or for whom insurance otherwise does not cover certain medications [21]. A recent systematic review showed the utility of PSPs in improving patient adherence to medications, as well as reducing healthcare utilization and costs [22]. For adalimumab, PSPs garnered significantly higher adherence, lower discontinuation rates, lower disease-related medical costs, lower all-cause medical costs, and lower rates of hospital visits [23,24]. While similar studies have not been conducted specifically for upadacitinib, multiple PSPs exist and could reasonably lead to similar outcomes in patients with AD. It is important for clinicians to have a discussion with patients regarding the importance of continuous AD treatment, especially when experiencing transitions or gaps in insurance coverage. PSPs provide a way to ensure continuation of treatment when insurance or cost barriers may impact access to care. Using PSPs to secure continuation of treatment could potentially prevent life-threatening complications such as those described in this case, maintaining disease control and quality of life.

## Conclusions

AD is a chronic inflammatory disease that predisposes to skin infections. When properly controlled, these infections rarely progress to systemic involvement. However, sepsis arising from poorly controlled AD remains a life-threatening yet preventable complication. Ensuring access to consistent therapy is key to reducing the risk of systemic infection. Novel therapies, including JAK inhibitors, offer promising treatment options for moderate-to-severe AD. However, their high cost significantly limits patient access, further exacerbated by transitions or gaps in insurance coverage. It is essential to ensure uninterrupted treatment for patients to maintain AD control and prevent life-threatening complications such as sepsis. Patient support programs can play a significant role in ensuring continuity of treatment when cost or insurance coverage presents barriers to access.
